# Insight into the structural, elastic and electronic properties of a new orthorhombic 6O-SiC polytype

**DOI:** 10.1038/s41598-020-64415-4

**Published:** 2020-05-05

**Authors:** Yassine El Mendili, Beate Orberger, Daniel Chateigner, Jean-François Bardeau, Stéphanie Gascoin, Sébastien Petit, Olivier Perez, Fouzia Khadraoui

**Affiliations:** 1 COMUE Normandie Université – Laboratoire ESITC - ESITC Caen, 1 Rue Pierre et Marie Curie, 14610 Epron, France; 2 CRISMAT-ENSICAEN, UMR CNRS 6508, Université de Caen Normandie, IUT Caen, Normandie Université, 6 boulevard Maréchal Juin, 14050 Caen, France; 3 GEOPS, Université Paris Saclay-Paris Sud, UMR 8148 (CNRS-UPS), Bât 504, 91405 Orsay, France; 4 IMMM, Le Mans Université, UMR6283 CNRS, Avenue Olivier Messiaen, 72085 Le Mans, France

**Keywords:** Electronic properties and materials, Structural materials

## Abstract

Different polytypes of SiC are described and predicted in literature. Here, we report the first occurrence of an orthorhombic 6O-SiC polytype as rock-forming mineral in the nickel laterite mine of Tiebaghi (New Caledonia). This new class of SiC crystallizes in the space group C*mc*2_1_ with 12 atoms per unit cell [a = 3.0778(6) Å, b = 5.335(2) Å, c = 15.1219(6) Å, α = 90°, β = 90°, γ = 120°]. The density of 6O-SiC is about 3.22 g/cm^3^ and the calculated indirect bandgap at room temperature of 3.56 eV is identical to 6H-SiC. Our results suggest that 6O-SiC is the intermediate state in the wurtzite to rocksalt transformation of 6H-SiC.

## Introduction

SiC is a highly attractive material for fabrication of microelectronic and optoelectronic devices due to its outstanding physical properties, such as a wide bandgap, high thermal conductivity coupled with low thermal expansion. The wide bandgap makes SiC a very attractive semiconductor to make devices for applications in high power, high frequency and high temperature environment^1–6^. In addition, its high strength gives this material exceptional thermal shock resistant properties and an exceptional hardness^4–6^.

A large number of SiC polytypes are already described and predicted in literature. The most common polytypes include 3C, 2H, 4H, 6H, 8H, 9R, 10H, 14H, 15R, 19R, 20H, 21H, and 24R^1,7,8^, where C, H and R mean cubic, hexagonal and rhombohedral symmetries, respectively. Natural SiC, known as moissanite, is extremely rare. Natural occurrences can be divided into three categories: kimberlites^9^; metasomatic rocks^10^; peridotites, serpentinites and podiform chromitites^11,12^.

**Figure 1:**
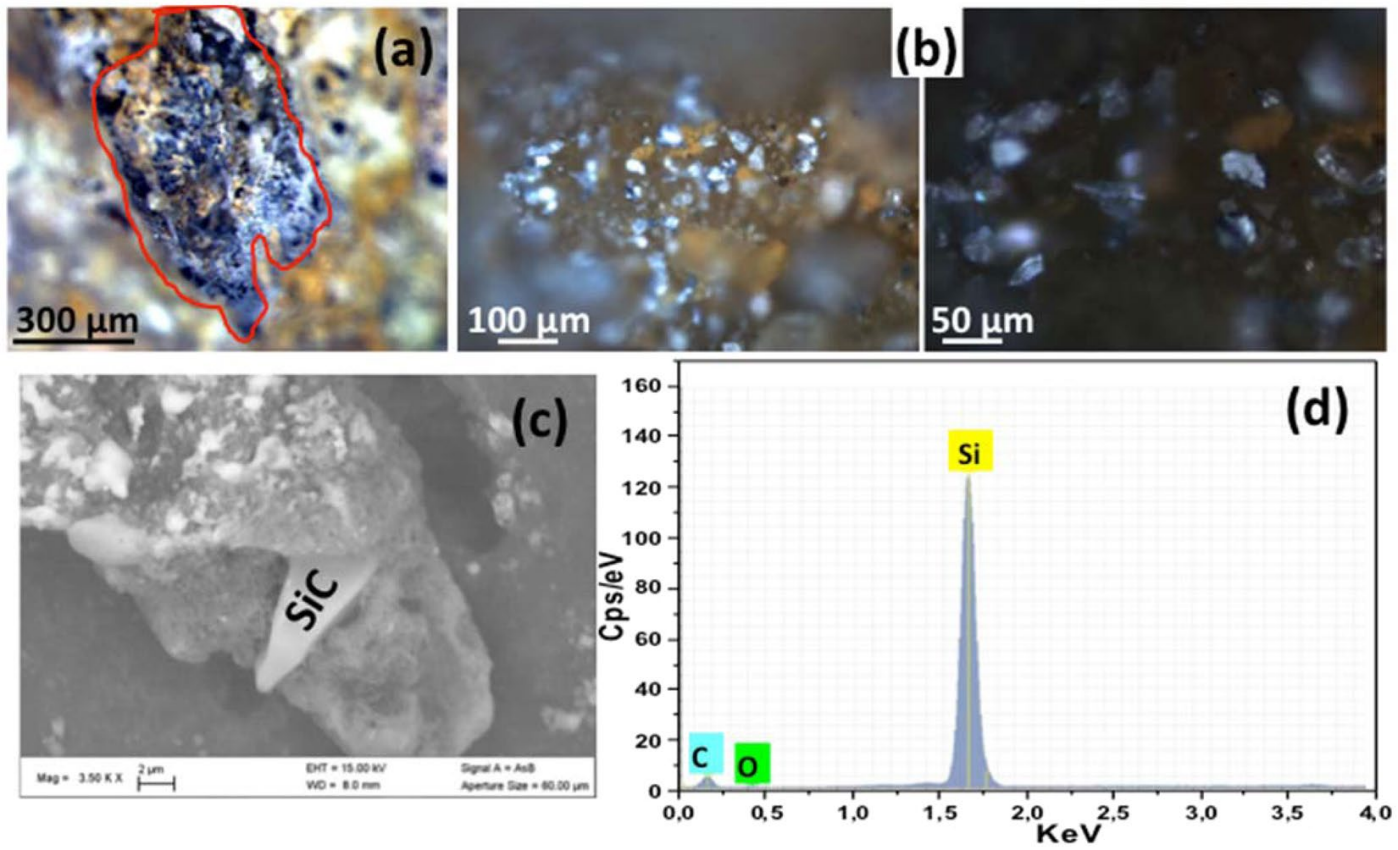
Porosities of siliceous breccia (**a)** Binocular image of siliceous breccia porosities, (**b)** Optical images of SiC present in porosities at two different magnifications (**c)** SEM image showing diamond cluster bearing-SiC extracted from breccia porosities and (**d)** EDS spectrum of SiC.

In this study, different grains of moissanite have been found and separated from the porosities of siliceous breccia originating from hydrofracturing of peridotites at Tiebaghi mine in New Caledonia (Fig. 1a). Moissanite occurs either as single grains or is associated with diamond in composite inclusions (Fig. 1c). These irregular fragments with a size of 10–75 μm exhibit various colors, including blue, green and yellow (Fig. 1b). Schmidt *et al*.^13^ have suggested that moissanite can be formed at very low temperatures during the alteration of ultramafic rocks, where hydrogen liberated by reactions involving serpentine would induce low oxygen fugacity, 6 to 8 log units below the Iron-Wustite buffer. However, SiC from peridotites and chromites in ophiolites are mainly subhedral to anhedral, and are similar to those observed in the siliceous breccia pores studied in this work. Rapid tectonic uplift would explain that these moissanite crystals are not decomposed to lower temperature phases. This finding suggests that these moissanite grains were liberated from the hostrocks and transported into the breccia pores^13,14^.

The structural, elastic and electronic properties of this new class of SiC were studied by single crystal X-rays diffraction, Raman spectroscopy and *ab initio* DFT calculation.

In this study, the optical microscopy analysis of the siliceous breccia porosities shows the presence of different crystals of moissanite. EDS analyzes confirm that all the moissanite crystals are mainly composed of C and Si (Fig. 1d).

## Structural properties of SiC

**Figure 2:**
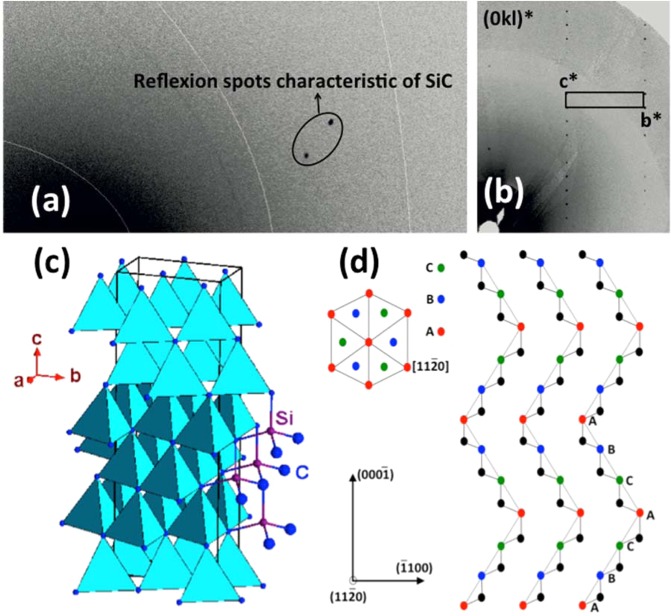
Single crystal diffraction experiment on 6O-SiC twinned single crystal. (**a)** one experimental frame exhibiting diffraction spots of SiC, (**b)** Part of the (0kl)* reciprocal plane of SiC assembled from the whole experimental frames. Reciprocal cell parameters and unit cell are drawn, (**c)** crystal structure refined in an orthorhombic 6O-SiC model and (**d)** stacking sequence of the 6O-SiC in the (1120) plane.

An extracted grain with a dark blue color and largest dimensions smaller than 10 ×10 ×10 μm^3^ was selected for SCXRD experiments (Fig. 2). A full data collection has been performed on a Rigaku Synergy S single crystal diffractometer equipped with a Cu micro focus X-ray source and a Dectris Eiger 1M photon counting detector. A first analysis of the full diffraction pattern has been done using the 6H-SiC polytype with the cell parameters *a* ≈ 3.078 Å, *b* ≈ 3.078 Å, *c* ≈ 15.13 Å, α = 90°, β = 90° and γ = 120°^15^; unindexed reflections inducing a doubling of the a and b periodicities are observed. The data integration was performed using Crysalispro [CrysAlisPro Software System, Version 1.171.39.46. Rigaku] and the new unit cell is a = 6.165(4) Å, b = 6.169(4) Å, c = 15.129(10) Å° α = 89.95(5)° β = 89.97(5)° γ = 119.96(7)°. An internal reliability factor (Rint) showed from the intensities of symmetry equivalent reflections that the deviation from the Laue classes 6/mmm, 6/m, −3, −3 m1 or −31 m is always larger than 52%. This excludes unambiguously the hexagonal or trigonal symmetries. The reciprocal space was then interprated considering the orthorhombic unit cell *a* = 3.0778(6) Å, *b* = 5.335(2) Å, *c* = 15.1219(6) Å, α = 90°, β = 90° and γ = 90° and twin components related by a three-fold axis parallel to *c*. The new integration leads to a Rint value of about 12% for the mmm Laue class. This value far to be perfect suggests an imperfect data quality certainly related to the tiny dimension of the crystal. Nevertheless, the structure has been determined by charge – flipping method^16^ with Superflip^17^ in the Cmc2_1_ space group and then refined using Jana2006^18^. Cmc2_1_ being non centrosymmetric, 3 additional twin domains have to be introduced. But only 3 twin domains related by a three fold axis have been found as significant from their respective intensities; the twin volume ratio are the following: 38(5)%, 36(5)% and 26(5)%.The final agreement factor is Rf = 0.0329(2) for 316 reflections with I ≥ 3σ(I) and 20 refinement parameters. A view of the structure is presented in Fig. 2c. The main structural characteristics of the 6H-SiC polytype are described as SiC_4_ tetrahedra sharing all their edges with their neighbours.

The crystal packing of 6O-SiC can be described by means of two alternating layers tilted with respect to one another by an angle close to 120°. However, slight differences can be noted compared to the hexagonal polytype. The 3 independent SiC_4_ groups are exhibiting distortions as evidenced by the Si-C bond lengths varying from 1.866(9) to 1.92(2) Å as well as by the Si-C-Si angles ranging from 107.8° to 111.2° which corresponds to deviations of nearly ±2° with respect to the values reported for cubic and hexagonal polyypes. This phenomenon is usually called planarization, a flattening of the tetrahedra correlated to an increase in bond angle. This leads to a non-centrosymmetric orthorhombic structure.

The structure can then be described by alternating a periodic stacking of blocks following the sequence …ABCACB… along the [0001] direction, illustrating the existence of six types of block boundaries (Fig. 2d). The atomic positions of the 6O-SiC polytype are given in Table 1.

**Table 1:** Fractional atomic coordinates and equivalent isotropic displacement parameters for the orthorhombic 6O-SiC refined structure.

Atom	Occupancy	x	y	z	U_iso_ (Å^2^)
Si1	1	0	0.8327(15)	0.858(1)	0.0050(11)
Si2	1	0.5	−0.0007(6)	0.5241(4)	0.0055(5)
Si3	1	0.5	0.6643(14)	0.69124(12)	0.0057(11)
C1	1	0	0.504(3)	0.6503(7)	0.0009(16)
C2	1	0	0.840(4)	0.9817(5)	0.0062(16)
C3	1	0.5	0.671(4)	0.8168(5)	0.0081(18)

In literature, 6H polytype is formed at very high temperatures (2300–2800 K) and at least at pressures of 3 GPa^19^. Because of the similarities between the polymorphism in SiC and ZnS, the transition models that have served as frameworks to study the wurtzite to rocksalt transition are the same for SiC. The high-pressure transition from the 6H wurtzite to the 3C rocksalt structure in SiC has been explored extensively with the help of computations^20–22^. It also has been observed in shock compression experiments at a pressure of 105 GPa^22^. It is found by *ab initio* calculations that 6H polytype under high temperature-pression undergoes a transition to the rocksalt structure through an orthorhombic intermediate state^20^. The space groups of the transition states are identified as *Pmm2* and *Imm2*^20^. Our results are particularly important because they suggest that the wurtzite to rock-salt phase transitions pass through the intermediate states with Cmc2_1_ symmetry. It is interesting to note that an intermediate orthorhombic phase with Cmc2_1_ symmetry has also been described in previous works for the same transition in different semiconductors such as GaN, InN, CdS and CdSe^23^. The finding of this new SiC structure being an intermediate phase between wurtzite and rock slat transition is in the line with the geodynamic context of the sample. Indeed, these SiC microcrystals occur together with diamond, and other reduced phases (Fe-Mn alloys, Al-rich chromite). These phases most likely crystallized in the lower mantle^24^. Second stage boninitic melts, described at Tiebaghi (New Caledonia)^25,26^, served as a natural elevator to transport these ultrahigh-pressure, reducing phases into the upper mantle. These resistant phases survived hydrothermal and supergene alteration.

## Elastic properties of SiC

The elastic constant tensors of 3C-, 6H- and 6O-SiC crystals, determined by Brillouin scattering, are listed in Table 2 along with available computed and experimental elastic constants. Using the second order elastic constants, the bulk modulus *B* is computed.

**Table 2:** Calculated elastic constants (*C*_*ij*_ in GPa) and bulk modulus (*B* in GPa) of 3C-, 6H and 6O- SiC.

Structures	Space group	Methods	*B*	*C*_11_	*C*_12_	*C*_13_	*C*_22_	*C*_23_	*C*_33_	*C*_44_	*C*_55_	*C*_66_	Reference
3C-SiC	F-43m	Theoretical	213	382	128	—	—	—	—	239	—	—	^41^
			214	406	118	—	—	—	—	255	—	—	This work
		Experimental	215	395	123	—	—	—	—	236	—	—	^42^
6H-SiC	P6_3_mc	Theoretical	214	470	103	50	—	—	493	168	—	—	^43^
			215	507	95	43	—	—	555	176	—	—	This work
		Experimental	220	501	111	52	—	—	553	163	—	—	^44^
6O-SiC	Cmc2_1_	Theoretical	215	507	95	43	507	43	555	176	176	206	This work

In general, the intrinsic mechanical stability of a solid is determined by certain conditions concerning the C_ij_ values related to the crystal symmetry. For an orthorhombic crystal, the independent elastic stiffness tensor reduces to nine components C_11_, C_22_, C_33_, C_44_, C_55_, C_66_, C_12_, C_13_ and C_23_ in the Voigt notation. The elastic components C_11_, C_22_ and C_33_ represent the elastic stiffness along the three-unit cell directions. The other constants are associated with either mixed behaviours (C_ij_’s with i ≠ j) or shears (C_44_, C_55_ and C_66_). The Born stability criteria^27^ for an orthorhombic system are:

1 $${\rm{B}}1={{\rm{C}}}_{11}+{{\rm{C}}}_{22}+{{\rm{C}}}_{33}+2({{\rm{C}}}_{12}+{{\rm{C}}}_{13}+{{\rm{C}}}_{23}) > 0$$

2 $${\rm{B}}2={{\rm{C}}}_{11}+{{\rm{C}}}_{22}-2{{\rm{C}}}_{12} > 0$$

3 $${\rm{B}}3={{\rm{C}}}_{11}+{{\rm{C}}}_{33}-2{{\rm{C}}}_{13} > 0$$

4 $${\rm{B}}4={{\rm{C}}}_{22}+{{\rm{C}}}_{33}-2{{\rm{C}}}_{23} > 0$$

The computed B1, B2, B3, and B4 values for the 6O-SiC are all positive with 1243, 824, 976, and 976 GPa, respectively. All the four conditions for mechanical stability are simultaneously satisfied and this clearly indicates that this orthorhombic polytype is a mechanically stable phase. C_44_ is an important parameter indirectly governing the indentation hardness of a material. The studied Cmc2_1_ structure possesses a large C_44_ value of 175 GPa, meaning its relatively strong strength against shear deformation. The bulk modulus *B* of 3C, 6H and 6O structures are similar (B ≈ 215 GPa), which is the indication of high hardness of these semiconductor materials. The C_33_ value is superior to C_11_ and C_22_ values, showing the compressibility along the *ab*-plane to be more pronounced than along the *c*-axis, towards a more anisotropic phase compared to 3C and 6H. Elastic constants yield the compressibility of the ***c*** axis smaller than the ***a*** and ***b*** axis (0.0018 versus 0.0021 and 0.0021 GPa^−1^).

## Vibrational properties of SiC

The Raman-active vibrational modes of our orthorhombic SiC were calculated through Density Functional Theory (DFT). For comparison, we also calculated the Raman spectra for cubic SiC and hexagonal 6H-SiC. 6H-SiC is among the most interesting SiC polytype for power electronic applications. Moreover, 6H-SiC exhibits high breakdown electric field, high thermal conductivity and a wide band gap of 3.1 eV at room temperature^4^. In addition, this polytype is the most abundant one occurring in terrestrial rocks.

**Figure 3:**
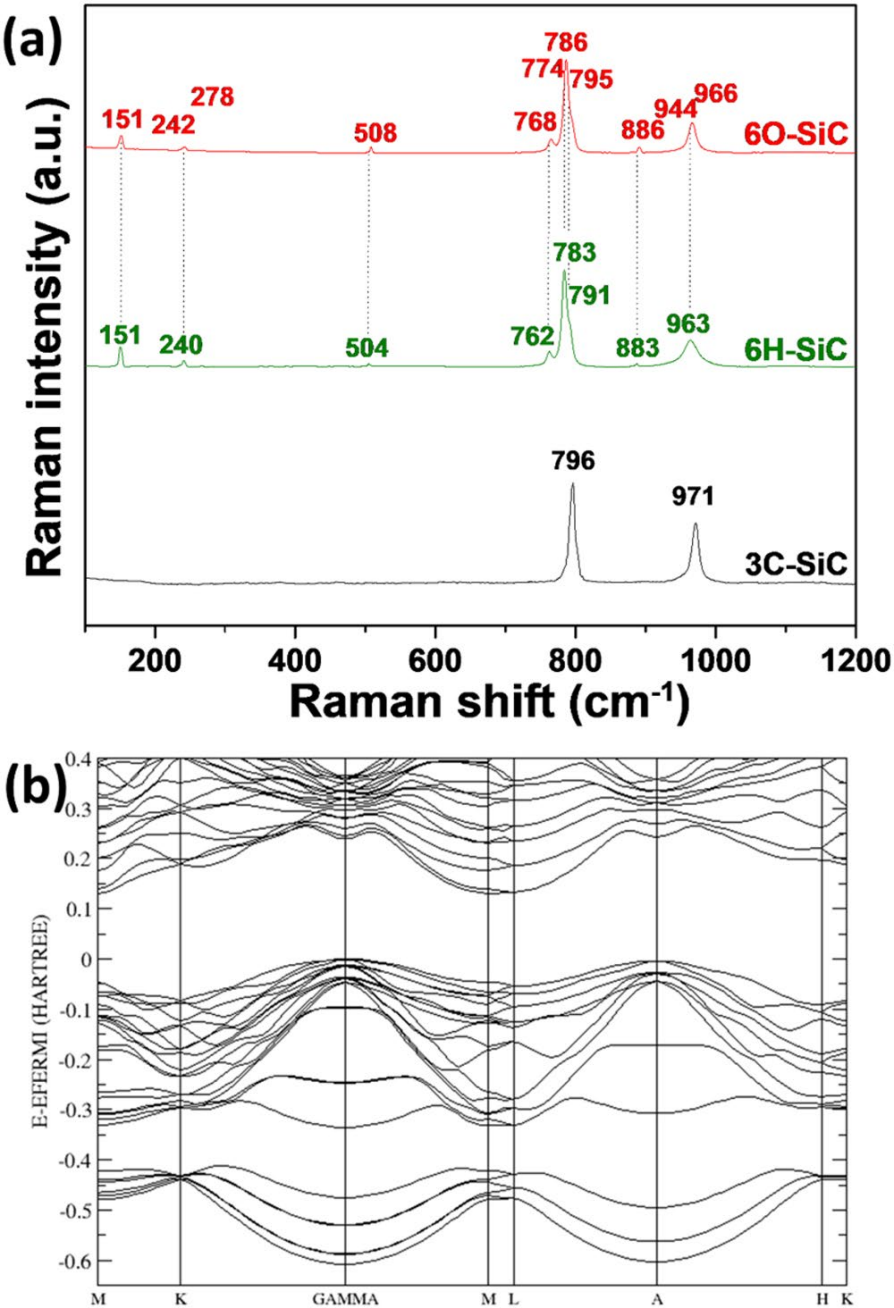
(**a**) Raman spectra of three different SiC crystals present in the siliceous porosities and (**b**) calculated electronic band structure of 6O-SiC.

The Raman spectra of SiC polytypes present in siliceous breccia pores are shown in Fig. 3a. We can detect three of the SiC polytypes: 3C, 6H and 6O.

References to standard transverse acoustic and optical phonons (TA and TO resp.), and longitudinal optical and acoustic phonons (LO and LA resp.) were obtained from literature for different polytypes^28^.

For 3C-SiC, only two strong Raman bands are observed at 972 cm^−1^ and 796 cm^−1^ assigned to the longitudinal E_1_(LO) and transverse A_1_(TO) optical phonon modes, respectively. For 6H-SiC, the experimental characteristic phonon modes are assigned as follows^28^: E_2_(TA) at 151 cm^−1^, E_1_(TA) at 240 cm^−1^, A_1_(LA) at 504 cm^−1^, E_2_(TO) at 762 and 783 cm^−1^, E_1_(TO) at 791 cm^−1^ and A_1_(LO) at 887 and 963 cm^−1^.

In the case of 6O-SiC, the main Raman modes were confirmed and matched with Raman-active vibrational modes in the orthorhombic C*mc*2_1_ structure. According to group theory, the orthorhombic C*mc*2_1_ structure with 12 atoms per unit cell gives rise to 33 Raman-active modes and factor group analysis gives irreducible mechanical representations for optical modes as Γ_optic_ = 11A_1_ + 6A_2_ + 11B_1_ + 5B_2_, which are all Raman active. Table 3 shows the vibrational modes of 6O-SiC obtained by DFT calculations and observed experimentally by Raman spectroscopy.

**Table 3:** Theoretical and experimental Raman-active vibrational modes of 6O-SiC. The symmetries are based on those calculated by DFT.

Polytype					
6O-SiC				6H-SiC	
Vibrational modes/Symmetries	DFT		Experimental (cm^−1^) ± 0.3	Vibrational modes/Symmetries^28^	Experimental (cm^−1^) ± 0.3
	Predicted wavenumber (cm^−1^)	Predicted intensity			
A_2_	150	vw	151 w	E_2_(TA)	151 w
A_1_	150.1	vw			
A_1_	152.9	vw			
A_2_	153.1	vw			
B_1_	245.6	vw			
B_2_	245.8	vw	242 vw	E_1_(TA)	240 vw
B_1_	249.7	vw			
B_2_	249.8	vw			
A_1_	278.8	vw			
A_2_	279	vw	278 vw	—	—
B_1_	281.7	—			
B_1_	283.8	—			
A_1_	509	vw	509 vw	A_1_(LA)	504 vw
A_1_	515	vw			
B_1_	618.9	—	Not observed	—	—
A_1_	761.5	m			
A_2_	762.7	m	**768 m**	E_2_(TO)	**762 m**
B_1_	765.5	vw			
B_2_	766.8	vw			
B_1_	774.5	vw	774 w	—	—
B_2_	775.4	vw			
A_1_	787.2	s			
A_2_	788	s	**786 s**	E_2_(TO)	**783 s**
A_1_	788.6	m			
A_1_	788.9	s			
A_2_	790	vw			
B_1_	797.8	m	**797 m**	E_1_(TO)	**791 m**
B_2_	798.8	m			
B_1_	838.7	—	Not observed	—	—
A_1_	885.9	vw	891 vw	A_1_(LO)	887 vw
A_1_	891.1	vw			
B_1_	943.8	vw	944 vw	—	—
B_1_	969.4	m	966 m	A_1_(LO)	963 m

vw: very weak, w- weak, m: medium and s: strong.

We can notice that the Raman spectral features of the 6H- and 6O-SiC polytypes are close to each other. This similarity confirms the results obtained by SCXRD meaning that the structural properties of these two polytypes are very close.

The pressure dependence of the Raman vibration modes in 6H-SiC was measured up to 95 GPa^29,30^. In these studies^29,30^, the optical modes are all shifted to higher wavenumbers with pressure. Consequently, based on the comparison between the 6O and 6H vibrational modes, we can attribute the shift of the main intense modes to a variation of pressure. The strongest optical modes A_1_ for 6O-SiC are present at 786 cm^−1^, while for 6H-SiC the strongest band E_2_(TO) is present at 783 cm^−1^. Similar observation can be made for the 2 other optical transverse modes of 6H, E_2_(TO) and E_1_(TO) present at 762 cm^−1^ and at 791 cm^−1^, respectively, shifted by about 5 cm^−1^ towards high wavenumbers in 6O-SiC. Then, according to the pressure dependence of the vibration modes wavenumbers^29,30^, the observed shifts correspond to an increase in isostatic pressure close to 1 GPa. Accordingly, this suggest**s** that the 6O polytype was formed at very high temperatures (2027–2527 °C) and at minimum pressures of 4 GPa.

## Electronic properties of SiC

In Fig. 3b, we show the calculated bulk band structure of 6O-SiC using the Density Functional Theory. The results show that 6O-SiC is an indirect band gap semiconductor with the top valence band located at Γ of the Brillouin zone and the conduction band edge along the *M*-*L* symmetry line. The calculated indirect bandgaps for 3C, 6H and 6O-SiC are 2.90, 3.56 eV and 3.56 eV, respectively (Table 4). We notice that our calculations underestimate the experimental bandgap of the 3C and 6H SiC^4^ polytypes. However, since the calculated indirect bandgap value of the 6O-SiC is equal to that of 6H polytype, we can reasonably expect that the real indirect bandgap of the 6O polytype is around 3.10 eV.

**Table 4:** Calculated and experimental energy gaps of the 3C, 6H and 6O structures. Experimental data are taken for SiC from reference ^4^.

Materials	Space group	*Energy gap (eV)*	
		Theoretical (this study)	Experimental
3C-SiC	F-43m	2.90 (indirect)	2.40 (indirect)
6H-SiC	P6_3_mc	3.56 (indirect)	3.10 (indirect)
6O-SiC	Cmc2_1_	3.56 (indirect)	—

In this study, we report the first occurrence of an orthorhombic 6O-SiC polytype as rock-forming mineral. 6O-SiC crystallizes in the space group C*mc*2_1_ with 12 atoms per unit cell. These findings establish a novel SiC mechanically stable phase with a density value close to that of 6H-SiC. The calculated indirect bandgap of 6H and 6O-SiC at room temperature are equals.

Among the SiC polytypes, 6H is the most easily synthetized and the best studied. For instance, 6H-SiC has been found to be very suitable substrates for GaN epitaxial layers for the deposition of laser diodes^31^.

The question that remains unresolved is if this new 6O-SiC structure can be also easily prepared, given its superior density? Most probably deposition as thin heteroepitaxial structures on single crystal substrates, known to stabilize films at large, GPa-like equivalent pressures, may allow future studies of this new phase.

## Methods

### Sample materials

Moissanite studied here naturally occurred in siliceous breccia rock (SB; SOLSA label: ER-NC00-0001) from Tiebaghi mine in New Caledonia. Tiebaghi is a mine and former village in Kaala-Gomen, New Caledonia, located on an ultramafic massif. The siliceous breccia samples are solid, porous beige rocks (pore size can reach cm scale). The breccia hosts lithoclasts of brownish-dark grey saprolite. Locally reddish spots occur, originating from weathering.

### SEM/EDS measurements

Imaging and compositional analysis were performed using scanning electron microscope JEOL JSM-6400 equipped with an X-ray energy dispersive system (SEM/EDS) operating at 20 kV and sensitive to elements with atomic numbers from Na to U.

### Micro-Raman measurements

The Raman spectra were recorded at room temperature using a DXR Raman microscope (Thermo Fisher Scientific, Inc., USA) equipped with a 900 lines/mm diffracting grating. The highest quality spectra (highest signal-to-noise) were obtained using green excitation (532 nm, Nd: YAG laser). Raman measurements were carried out at low laser power (approximately 1 mW at the sample surface) to avoid unintentionally heating alteration, structural modifications or phase transitions. A 100x magnification long working distance objective was used to focus the laser onto the sample and collect the scattered light in a backscattering geometry. Raw data was collected over a range of 80–2200 cm^−1^. The spectral region 3200–4000 cm^−1^ was investigated for some minerals species when OH stretching vibration modes were potentially expected. The spot diameter of the laser was estimated at 0.6 μm. Raman spectra were systematically recorded twice with an integration time of 60 s. For each mineral, 5 spectra were recorded and averaged. Peak deconvolution and integration were performed with the Origin software using the Gaussian-Lorentzian function.

### SCXRD measurements

Single crystal X-ray diffraction. A Synergy S Rigaku 4-circles diffractometer equiped with Cu micro focus source and an Eiger 1M Dectric detector has been used to analyze the SiC grains. A full data collection was performed on the highest quality sample. The analysis of the reciprocal space is done using *Crysalis*^*Pro*^ software^32^. The structure has been solved using Superflip software^17^ and refined with Jana2006^18^.

### Computational details

The Raman-active vibrational modes of 60-SiC were calculated through Density Functional Theory (DFT) as implemented in the Crystal17 package^33^. The B3LYP approximation was used and the wave functions were expanded as a linear combination of Bloch functions using Gaussian-type atomic orbitals (6–31 G*)^34^. The Coulomb and exchange series were truncated with overlap thresholds of 10^−8^, 10^−8^, 10^−8^, 10^−8^ and 10^−16^, A Monkhorst-Pack sampling scheme of 16 × 16 × 16 for cubic SiC, 16 × 16 × 10 for hexagonal 6H-SiC and orthorhombic SiC were chosen after some tests. The convergence threshold on energy for the self-consistent-field procedure was fixed at 10^−10^ Hartree for structural optimizations and for vibration frequency calculations. Frequencies at the Gamma point were evaluated within the harmonic approximation as implemented in Crystal17^35,36^. The Raman intensities of the vibrational modes were calculated analytically by exploiting the scheme described by Maschio *et al*.^37,38^ at ambient temperature and an exciting wavelength of 532 nm. This scheme is based on the solutions of first and second-order coupled Perturbed–Hartree–Fock/Kohn–Sham (CPHF/KS) equations^39,40^. For the calculation of the elastic constants, we have used the default settings on size of lattice deformation (0.01 Å) and 3 points, including the central point with zero displacement for the numerical second derivatives calculations. We have used the default energy and force convergence criteria for vibrational frequency and elastic constants calculations as defined by Crystal17. These convergence criteria are typically tighter than separate. The band structure and DOS of the models were constructed along the appropriate high-symmetry directions of the corresponding irreducible Brillouin zone.

## Data Availability

The experimental and computational data that support the findings of this study are available from the corresponding author upon request.
